# Computational Investigations to Identify Potent Natural Flavonoid Inhibitors of the Nonstructural Protein (NSP) 16/10 Complex Against Coronavirus

**DOI:** 10.7759/cureus.68098

**Published:** 2024-08-29

**Authors:** Silambarasan Tamil Selvan, Gurupavan Kumar Ganta

**Affiliations:** 1 Center for Global Health Research, Saveetha Medical College and Hospitals, Saveetha Institute of Medical and Technical Sciences (SIMATS) Saveetha University, Chennai, IND; 2 Biochemistry, Saveetha Medical College and Hospitals, Saveetha Institute of Medical and Technical Sciences (SIMATS) Saveetha University, Chennai, IND

**Keywords:** coronavirus, molecular dynamics simulation, molecular docking, in silico, flavonoids

## Abstract

Introduction: Globally, the viral pandemic has spread rapidly, resulting in widespread infections. The coronavirus family (CoVs) is one of the various viral families capable of infecting mammals, causing diseases related to the gastrointestinal, neurological, and respiratory systems. Flavonoid compounds have been identified as potentially effective antiviral agents, specifically targeting the virus's nonstructural protein (NSP) 16/10. Flavonoids have also been shown to inhibit virus replication and viral attachment to host cells, making them a promising candidate for antiviral treatment. Further research is needed to understand the full potential of flavonoids as antiviral agents.

Methodology:This study investigated natural compounds derived from medicinal plants using in silico screening. In addition to assessing drug-likeness, pharmacokinetics, docking, molecular dynamics simulation, bioavailability assessment, and exploration of molecular targets, the screening process entailed analyses of molecular targets and bioavailability. The molecular properties and potential antiviral efficacy of these phytochemical candidates were determined by analyzing them as drug candidates. The results of the study showed that these compounds had potential antiviral activity and could be developed as therapeutic agents. Furthermore, the study showed that the compounds had good bioavailability, suggesting that they are suitable for use as therapeutic agents.

Result: An in silico method was used to identify flavonoid compounds for potent antiviral drug molecules against the coronavirus protein complex NSP16/10 protein. The NSP16/10 complex protein binding energy values were -6.14 for isoquercetin, -6.902 for narirutin, -6.052 for myricetin, -7.10 for hesperidin, -4.392 for silibinin, -3.997 for baicalein, -3.712 for taxifolin, and -3.321 for petunidin. Molecular dynamics simulations showed that isoquercetin, hesperidin, and narirutin flavonoids interacted with the COVID-19 virus protein complex NSP16/10 protease up to 100 nanoseconds.

## Introduction

Coronaviruses (CoVs) are a group of viruses that cause infection in many species of animals. In addition to causing respiratory, neurological, and gastrointestinal disorders, they can spread to humans. In the past, two pandemic outbreaks caused serious infections in people, including severe acute respiratory syndrome coronavirus (SARS-CoV) and Middle East respiratory syndrome coronavirus (MERS-CoV) [1]. In the same way, the severe acute respiratory syndrome coronavirus-2 (SARS-CoV-2) causes viral pneumonia, causing significant concerns for global health [2,3]. A worldwide alarm has been raised regarding human health, and there is an urgent need to discover or invent a suitable treatment for the disease. CoV produces positive-stranded, one-stranded RNA viruses with large viral genomes. The genomes of CoVs do not differ from those of other beta-corona viruses (SARS-CoV-2) in that they encode an untranslated region (UTR), nonstructural proteins (NSPS), and a replicase complex (orf1ab). SARS-CoV-2 is a beta-coronavirus, and it differs from MERS-CoV and SARS-CoV [3,4]. Coronavirus genes share 89.1% nucleotide gene similarity with the SARS-CoV viral genome. The CoV RNA genome is replicated and transcribed using a dynamical RNA replication mechanism comprising a chain of 16 nonstructural viral proteins. The NSP16 of SARS-CoV and MERS-CoV also stimulates methyltransferase activity at the ribose site at the beginning of translation to accelerate methylation [5,6]. It activates ribose 2′-O-methyltransferase when it forms a heterodimer with NSP10 and acts as a cofactor. As a result of NSP16/10, the CoVs virus's genomic components develop more familiarity with human RNA as it augments the 2′-O-methyltransferase function [7,8]. Virus RNA is protected by methyltransferase enzymes from 50-30 exoribonucleases breaking down, ensuring successful translation and preventing human immune recognition [9,10].

There are various potential bioactive compounds in South Indian herbal medicine, including flavonoids, polyphenols, terpenoids, phenolic acids, anthocyanins, and tannins. In addition to their antiproliferative, anti-inflammatory, antiatherogenic, antiallergic, antimicrobial, immunomodulatory, and antiviral activities, these compounds are also able to show antiproliferative, anti-inflammatory, antiatherogenic, immunomodulatory, and antiviral activities [11-14]. Natural compounds such as these are not only non-toxic but also environmentally friendly. Flavonoids derived from medicinal plants have been shown to have antiviral activity against viruses such as H1N1, Ebola, influenza, and dengue [15]. There has been renewed interest in flavonoids as treatment options for the coronavirus pandemic. Flavonoids have a crucial function in coronavirus treatment by either blocking virus binding to human cell receptors or targeting host-specific receptors, therefore suppressing virus entry into cells; (ii) inhibiting RNA synthesis and replication; and (iii) reducing the virulence of the virus to restore the body's innate immunity. A viral invasion triggers an inflammatory response that can be interrupted by these approaches [15]. Natural medicinal plants have been shown to inhibit viral proteins, making them an attractive therapeutic option for viral infections. There are many advantages to the use of these plants, including their effectiveness at low concentrations, their ease of accessibility in nature, and their minimal to no adverse reactions.

This study aims to discover promising antiviral drugs from flavonoid compounds derived from natural medicinal plants. Several compounds are currently being investigated for their ability to suppress coronavirus replication and protein (NS16/10) and confirmed through various analyses, including molecular docking and molecular dynamics (MD) simulations.

## Materials and methods

Collection of protein data

The coronavirus (COVID-19) protein NSP16/10 was obtained from the protein database (PDB). A protein complex's ID was extracted, and water molecules and other elements were removed from PDB data. Furthermore, we saved the PDB file for future research purposes.

Selection and preparation of ligands

In this study, the eight flavonoid compounds were selected from medicinal plants, and the structure was retrieved using the PubChem database, namely, taxifolin, narirutin, hesperidin, baicalein, isoquercetin, myricetin, petunidin, and silibinin. The 3D molecular structures were downloaded as SDFs (spatial data files). Modifying the build module of Avogadro 1.2.0, polar hydrogen atoms were added into the ligands at pH 7.4. Geometry optimization and energy minimization in Avogadro were performed using the MMFF94 force field and the steepest descent algorithm. PDB files were created from the optimized structures. These files will be used for further analysis. The ligands were corrected for torsion, and polar hydrogens were added.

Protein preparation

The protein preparation involved removing crystallographic water molecules and ions during the import process. In the MVD workspace, both the ligands and target proteins were processed using the Protein Preparation Wizard. Subsequently, active sites (cavities) within the target protein were identified and detected, with the settings adjusted to detect up to five cavities, a grid resolution of 0.80, and a probe size of 1.2. The docking scores showed significant hydrogen bonding and interactions between the ligands and key amino acids within the target protein binding pocket. The structures were then screened against predicted structures of COVID-19 NSP16/10 complex proteases using Maestro v11 (Schrödinger, United States). This protein was optimized using Schrödinger Protein Preparation Wizard at neutral pH. The active site residues for each NSP16/10 protease were determined using Glide v7.1 (Schrödinger, United States) after the protein structures were built using liquid-simulated force field potentials. The LigPrep module was used to modify ligands to be chemically accurate, including adjusting for protonation, stereochemical variations, ionization states, and energy minimization.

Analysis of molecular docking

The NSP16/10 protein of the coronavirus was docked using Schrödinger software by targeting selected ligands at the active site. The proteins were prepared by adding polar hydrogen atoms to amino acid atoms and assigning Gasteiger charges for protonation types. Molecular water (H_2_O) was eliminated from both protein and ligand structures. Receptor bins were set at -0.28 to 5.55 Å, outline bins at -0.22 to 4.44 Å, and the ligand and receptor spheres at 1.35 to 1.55 Å. NSP16/10 protein maintained their active sites during docking outside these parameters. The binding hit and docking score competence of the compounds was evaluated and recorded.

MD simulations

The MD simulations were conducted using Schrödinger Desmond v4.2 software to evaluate the binding energy, interactions, and stability of potential flavonoid compounds with the receptor. Specifically, we ran 100-nanosecond (ns) simulations with explicit solvent molecules. Water molecules and ions were selected to solvate the complexes, and energy minimization and periodic boundary conditions were applied with an acceptable limit of 1000 kJ/mol/nm. The results of the MD simulations for the protein-ligand complexes were analyzed and documented [16].

## Results

Ligand selection and preparation

Molecular docking was performed on eight flavonoid ligand compounds: taxifolin, narirutin, hesperidin, baicalein, isoquercetin, myricetin, petunidin, and silibinin. The 2D structures were obtained from the PubChem database in SDF format. The strongest binding was observed for hesperidin, followed by narirutin and myricetin, with the NSP16/10 complex protein of coronavirus. Additionally, hesperidin demonstrated remarkable binding affinity for the coronavirus NSP16/10 protein, followed by narirutin, isoquercetin, taxifolin, and petunidin (Table 1).

**Table 1:** Physicochemical properties of flavonoid compounds.

Compound Name	Molecular Formula	Molecular Weight (g/mol)	Compound Function	Plant Source
Isoquercetin	C_21_H_20_O_12_	464.4	Hepatoprotective, anti-inflammatory, antimicrobial, antifungal, antigenotoxic, anti-viral activities	Glycyrrhiza glabra
Narirutin	C_27_H_32_O_14_	580.5	Anti-inflammatory, antimicrobial, antifungal, anti-viral activities	Citrus medica
Myricetin	C_15_H_10_O_8_	318.23	Anti-inflammatory, antimicrobial, antigenotoxic, anti-viral, anti-diabetic activities	*Epilobium hirsutum*,* Tetraclinis articulata*
Hesperidin	C_28_H_34_O_15_	610.6	Antiviral, anti-inflammatory, antifungal, neuroprotective, anti-diabetic activities	Centaurea furfuracea
Silibinin	C_25_H_22_O_10_	482.4	Anti-inflammatory, antimicrobial, anti-viral, anti-diabetic activities	Silybum marianum
Baicalein	C_15_H_10_O_5_	270.24	Antiviral, anti-inflammatory, antifungal, neuroprotective, anti-diabetic activities	Scutellaria baicalensis
Taxifolin	C_15_H_12_O_7_	304.25	Anti-inflammatory, antimicrobial, antifungal, antigenotoxic, anti-viral, anti-diabetic activities	Cedrus brevifolia
Petunidin	C_16_H_13_O_7_	317.27	Antioxidant, anti-inflammatory, anti-viral activities	Acacia nilotica

Molecular docking studies

**Figure 1:**
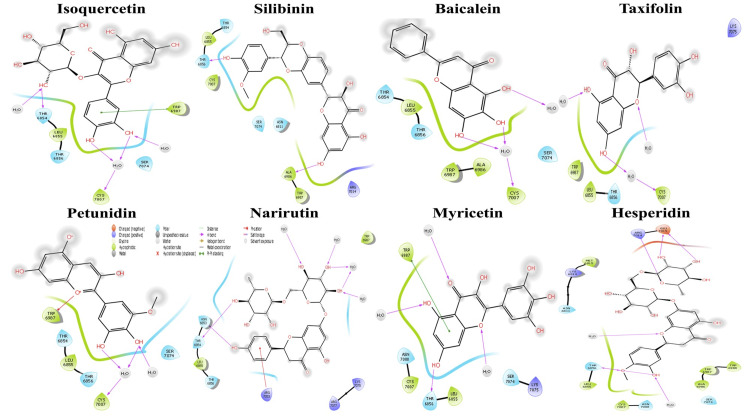
Molecular docking of the NSP16/10 complex protease protein against selected ligands and 2D interaction of the residues (Schrödinger software, JSS University, Ooty).

Molecular docking studies examined the binding affinity of both ligands and active residues within the protein's binding cavity. To determine the active residues located in the protein's binding pocket, a grid size of 3 Å was fixed relative to the native ligands of NSP16/10. The selection criteria were based on the function of optimal interactions, prioritizing the docking score (Table 2). Among the eight flavonoids tested, the highest binding energy (-7.10 kcal/mol) was observed against NSP16/10. In particular, the docking score for the NSP16/10 complex ranged from -7.10 to -3.32 (Figure 1). The binding energy score values were -6.14 for isoquercetin, -6.902 for narirutin, -6.052 for myricetin, -7.10 for hesperidin, -4.392 for silibinin, -3.997 for baicalein, -3.712 for taxifolin, and -3.321 for petunidin. Among the tested compounds, petunidin produces the lowest docking score (-3.32 kcal/mol) against the NSP16/10 complex. The narirutin binds to the active residues by showing six bonds, including five hydrogen bonds (THR 6854, CYS 7007, and SER 7074) and one pi-pi stacking (TRP 6987).

**Table 2:** Interactive profiles of flavonoid compounds with active site residues of target proteins.

Compound	Residue	Interaction	Type of Bond
NSP16/10 complex protease			
Hesperidin	GLY 143	O	H bond
	WATER	OH	H bond
	GLU 166	OH	H bond
	ARG 188	OH	H bond
Silibinin	ASN 142	OH	H bond
	CYS 143	OH	H bond
	THR 190	OH	H bond
Taxifolin	GLU 166	OH	H bond
	HIE 41	H_2_O	H bond
Petunidin	CYS 145	OH	H bond
	GLN 192	OH	H bond
	THR 190	OH	H bond
Narirutin	GLU 166	OH	H bond
	ARG 188	OH	H bond
	ASP 187	OH	H bond
	ASP 187	H_2_O	H bond
Isoquercetin	GLN 192	OH	H bond
	THR 190	OH	H bond
	THR 190	OH	H bond
	GLU 166	OH	H bond
	GLU 166	OH	H bond
	GLU 166	O	H bond
	HIE 41	-	Pi-Pi
Baicalein	GLU 166	OH	H bond
	HIE 163	OH	H bond
	HIE 41	-	Pi-Pi
Myricetin	ARG 188	OH	-
	HIE 163	OH	-

MD simulation

**Figure 2:**
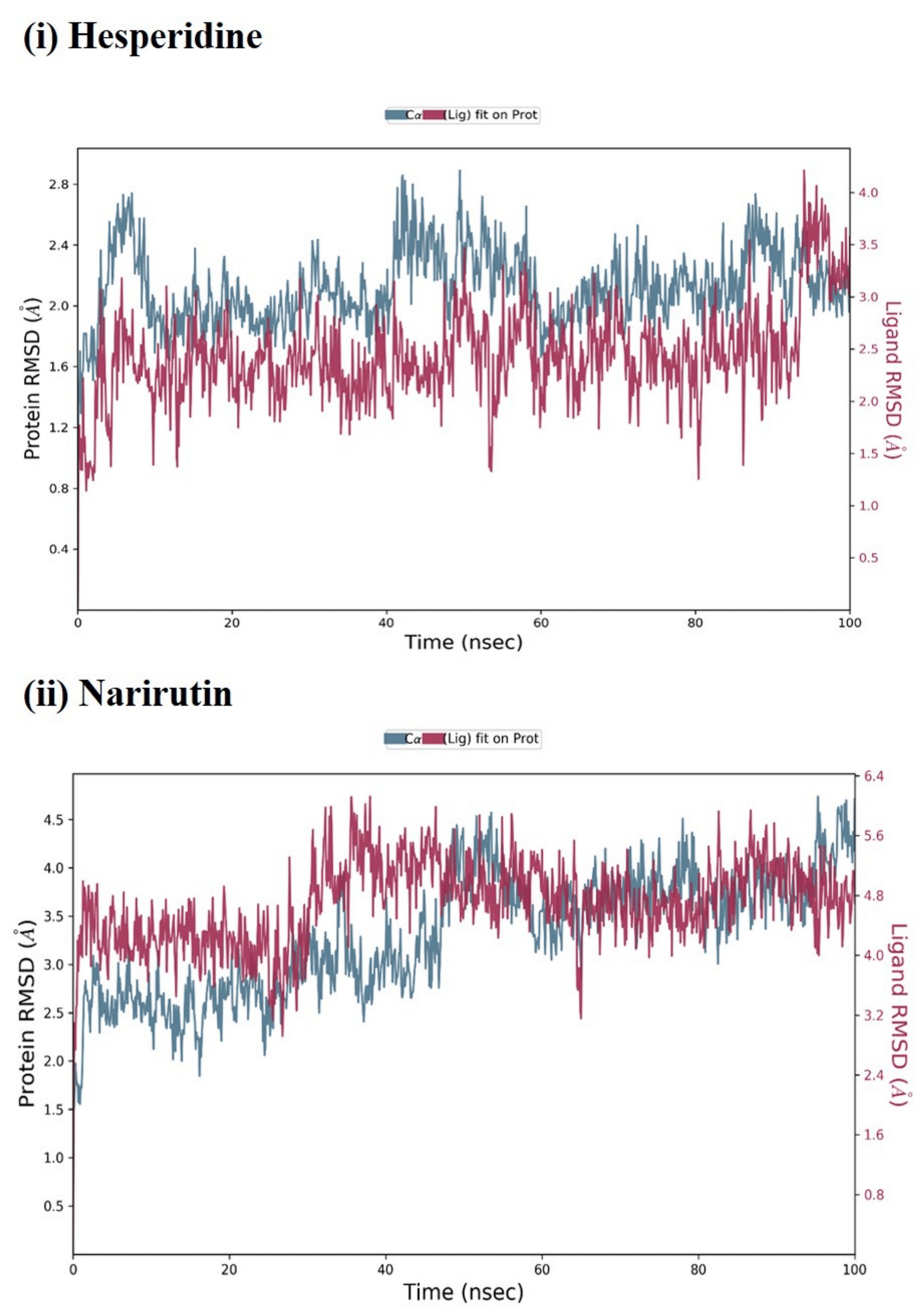
Analysis of molecular dynamics for the NSP16/10 complex protein and selected compounds (i) hesperidin and (ii) narirutin at 100 ns.

The MD simulations were performed for the docked complexes of hesperidin and narirutin for a period of 100 ns. The MD simulation showed that the ligand binds stably to the NSP16/10 protein of active site residues (Figure 2). The MD results showed in NSP16/10 protein residues, low root mean square deviation (RMSD) changes get correlated with docking studies based on the interaction of residues such as Asn6853, Thr6854, Thr6856, Trp6987, and Cys7007. This reveals the better binding nature of both flavonoid compounds hesperidin and narirutin to the active site of the NSP16/10 protein.

## Discussion

COVID-19 is currently combated with vaccines derived from four major SARS-CoV-2 structural proteins: nucleocapsid protein, spike glycoprotein, membrane glycoprotein, and small envelope glycoprotein. Antibodies designed to recognize and fight the virus are produced by the vaccines as a result of stimulating the immune system. Pandemic vaccines are being distributed throughout the world. Meanwhile, the emergence of various mutated SARS-CoV-2 variants could diminish the efficacy of these vaccinations, underscoring the necessity for innovative therapeutic strategies to address COVID-19 [17]. The emergence of a new variant of this virus may necessitate the implementation of distinct approaches, such as developing new vaccines, treatments, and diagnostic tools. Additionally, research is underway to develop more effective surveillance and monitoring techniques for new variants.

NSP16 is a promising target because it acts as a 2'-O-methyltransferase enzyme, altering viral mRNA from cap-0 to cap-1 in order to evade the host's immune system. By inhibiting the enzyme's activity, the virus' replication cycle may be disrupted, and its spread may be decreased. Consequently, NSP16 may be a promising target for antiviral drugs. The NSP16 enzyme is essential for COVID-19's RNA stability and can be inhibited therapeutically [18]. In addition to NSP16 being a good target for vaccine development, it may also be a good candidate for blocking viruses from replicating by causing antibodies to bind and recognize the enzyme. The in silico screening and molecular docking techniques we used in our study allowed us to identify eight flavonoid bioactive compounds extracted from medicinal plants. Inflammation can be reduced, oxidative damage reduced, and tumor growth can be inhibited by these compounds. Developing these compounds into natural cancer treatment drugs is possible. In addition, these compounds can be combined with other drugs to improve their efficacy.

Several bioactive compounds isolated from medicinal plants are potential drug candidates for viral and respiratory diseases. Bioactive compounds inhibit SARS-CoV and CoV viral replication and release [19]. In addition to decreasing inflammation, these compounds have also been shown to scavenge oxidative stress, which is important for respiratory disease pathogenesis. Furthermore, they are proven to reduce respiratory disease-related mortality and morbidity. A study by Ho et al. showed that plant compounds could block the direct binding of SARS-CoV S-proteins and pseudo-typed retroviruses. Hesperidin and narirutin bioactive compounds from medicinal plants may inhibit key proteins and enzymes of SARS-CoV-2, suggesting that new therapeutic agents for treating COVID-19 may be developed based on these findings [19]. Moreover, these bioactive compounds have been shown to possess antiviral properties against the SARS-CoV-1 virus and may also inhibit SARS-CoV-2 replication.

This study examined the antiviral properties of hesperidin and narirutin, naturally occurring compounds found in medicinal plants. Our screening results indicated that two bioactive compounds are potentially useful as drugs based on molecular docking sites. This will streamline the drug discovery process [20]. Hesperidin showed more antiviral activity than narirutin, but both compounds showed strong antiviral properties. Furthermore, the compounds showed low toxicity. The potential for them as antiviral drugs needs to be further explored. The present work employed the Glide v7.1 (Schrödinger, United States) program to predict the potential binding affinity of the phytocompounds hesperidin and narirutin against the NSP16/10 protein from SARS-CoV-2 after identifying them based on the drug-likeness criteria. A docking analysis was then performed to reveal the mode of binding and interactions between the two phytochemicals. The docking simulations showed that both phytochemicals strongly bound NSP16/10. MD computational simulations were conducted in order to validate the binding energy of the two phytochemicals. As demonstrated by the docking scores for hesperidin (-7.1 kcal/mol) and narirutin (-6.902 kcal/mol), these compounds bind efficiently to the active site of the NSP16/10 protein of SARS-CoV-2.

In the study by Chaturvedi et al., natural phytochemicals such as fagaronine, isoboldine, sageone, lycorine, and wogonin were found to be potential inhibitors, whereas a docking study showed that the target had significant binding energy against the coronavirus main protease, which was 6.21, 5.99, 5.97, 5.86, and 5.62 kcal/mol [20]. Shi et al. reported that andrographolide and its fluorescent derivative (nitrobenzoxadiazole-conjugated andrographolide) inhibited the activities of the main protease (Mpro) of both SARS-CoV and SARS-CoV-2 [21]. Similarly, research by Krafcikova et al. has shown that six amino acid residues (Asp99, Asn101, Asn43, Asp130, Lys170, and Asp114) in the active site of SARS-CoV-2 NSP16 are absolutely conserved. To inhibit RNA methylation in coronaviruses, it is crucial to prevent the interaction of these residues [22]. Notably, the two natural compounds, hesperidin and narirutin, can form hydrogen interactions with some of these residues [22].

In addition, Chen et al. showed that a quercetin derivative (quercetin-3-β-galactoside) inhibited the SARS-CoV 3C-like protease (3CLPro) activity. In a study by Shahdam et al., artepillin C, ellagic acid, hesperetin, kaempferol, and quercetin were tested against the RNA polymerase and Mpro of SARS coronavirus with binding energies ranging from -7.8 to -5.3 kcal/mol, respectively [23]. The conformational stability of hesperidin and narirutin molecules with the Mpro was validated by MD simulations, which showed that the molecules adopted a relatively stable conformation when they interacted with the enzyme [24]. Additionally, the binding energy between the molecules and enzyme further confirmed the stability of the reaction. Hesperidin exhibited a higher binding affinity than narirutin in the simulations as well. These ligands displayed minor variations in their RMSDs with each protein conformation over 100 ns.

Hossain et al. reported that the Mpro molecule fluctuated between 45 and 67 ns but later returned to an equilibrium state. The Mpro-catechin gallate complex showed a high fluctuation between 8 ns and 35 ns but then reached a stable position. Initially, the complex containing 3-O-malonylglucoside of Mpro and quercetin was stable. However, it fluctuated considerably after 45 ns [25]. A study by Mohanasundaram et al. reported RMSD values for glycyrrhizin, rutin, and violaxanthin molecules of 0.19 nm, 0.18 nm, and 0.18 nm, respectively, and for 0.2 nm, 0.21 nm, and 0.2 nm. In the radius of gyration (total and around axes), the stability of 3CL with glycyrrhizin, rutin, and violaxanthin is shown at 2.35 Rg (nm) up to 20,000 ps [26]. Consequently, the ligands were able to stably bind to the enzyme without exhibiting any structural variations. The simulations showed that the ligands were more suitable for use as inhibitors since they had a lower binding energy. Three different ligands that bind well to NSP16/10, hesperidin and narirutin, did not cause conformational instability. Based on the simulation results, Hesperidin and narirutin may be potential inhibitors of NSP16/10.

Further studies are required to investigate the potential of these ligands as inhibitors. The RMSF (root mean square fluctuation) analysis indicated that the ligands maintained the integrity of the NSP16/10 protein loop regions. The time development for NSP16/10 when subjected to various strengths and directions of electric fields. MD modeling showed a stable protein-ligand association, while the protein backbone showed only slight variations.

This study has limitations due to its reliance on in silico methods, which may not fully replicate in vivo conditions. Hesperidin and narirutin must be validated for their efficacy and safety through clinical and experimental trials. The present study extensively investigated drug interactions and potential off-target effects using an in silico approach. The study also considered the possible development of virus resistance. In addition, the bioavailability and pharmacokinetics of the compounds in humans remain complex.

## Conclusions

In this study, flavonoid compounds were shown to be effective as antiviral agents against the COVID-19 virus by targeting the NSP16/10 protein complex. Several flavonoids were identified as promising antiviral agents by conducting a series of in silico screening procedures, including drug-likeness, pharmacokinetics, docking, MD simulations, and bioavailability assessments. Docking of COVID-19 NSP16/10 protease complex proteins with eight compounds was conducted. Docking scores on hesperidin (-7.10 kcal/mol) and narirutin (-6.902 kcal/mol) were both high for the NSP16/10 complex. An NSP16/10 protease simulation showed that isoquercetin, hesperidin, and narirutin exhibited high binding affinities and stable interactions over a 100 ns time frame. In addition, these flavonoids, particularly isoquercetin, hesperidin, and narirutin, show potential for further development as COVID-19 therapeutic agents. Further experimental validation and clinical trials are needed to confirm these drugs' efficacy and safety.

The present study reports the use of plant-mediated flavonoid compounds to evaluate antiviral (COVID-19) activity and provide alternative drug compounds to control viral replication prevalence. Therefore, those plant flavonoid derivatives could be fabricated as innovative formulations for natural drugs and for various biological applications in the near future.
